# Context-Dependent Function of Myoepithelial Cells in Breast Morphogenesis and Neoplasia

**DOI:** 10.1007/s40610-015-0027-x

**Published:** 2015-10-05

**Authors:** Saevar Ingthorsson, Bylgja Hilmarsdottir, Jennifer Kricker, Magnus Karl Magnusson, Thorarinn Gudjonsson

**Affiliations:** 1 Stem Cell Research Unit, BioMedical Center, Faculty of Medicine, University of Iceland, Reykjavik, Iceland; 2 Department of Laboratory Hematology, Landspítali-University Hospital, Reykjavik, Iceland

**Keywords:** Myoepithelial cells, Luminal epithelial cells, Breast cancer, Breast morphogenesis, Basal cells, EMT

## Abstract

Myoepithelial cells (MEPs) are specialized cells derived from epithelial progenitor cells, yet they also express the contractile machinery of smooth muscle cells. MEPs are prominent in glandular tissues where their function is to help expel secretions generated by the glandular epithelial cells. In the breast, MEPs are part of the bi-layered breast epithelium that line ducts and alveoli positioned perpendicular to the luminal epithelial cells (LEPs), separated from the surrounding stroma by the basement membrane. Researchers have recognized MEPs as important regulators of structural and functional behavior of LEPs, namely having role in polarization of LEPs, and regulating milk production. Furthermore, they have also been proposed to act as tumor suppressors as their presence inhibits invasion of cancer cells into the surrounding stroma. There is, however, accumulating evidence that MEPs in normal breast, carcinoma in situ and in invasive breast cancer differ significantly in terms of marker expression and this may truly interfere with their ability to behave as tumor suppressors. The term myoepithelial cell is often used synonymously with basal cell. While all MEPs, due to their position, can be referred to as basal cells, some basal cells do not fulfill the criteria of being MEPs. Synonymous use of these terms may hold true under normal conditions but careful interpretation of these terms should be used in breast cancer. In recent years, partial myoepithelial differentiation and epithelial to mesenchymal transition (EMT) have been shown to be associated with, and in some cases, necessary for cancer invasion and metastasis. In this review, we will discuss the context-dependent role of MEPs in breast morphogenesis, tumor suppression, and also the appearance of basal or partial myoepithelial differentiation in aggressive forms of breast cancer.

## Introduction

Exocrine glands such as the salivary glands, the submucosal glands in the upper airways, and the breast glands need contractile elements to help the secretory luminal epithelial cells (LEPs) to move glandular secretions out towards the surface. These contractile elements are myoepithelial cells (MEPs), a cell type of epithelial origin that has acquired partial mesenchymal traits and contractile functions [1–3]. Understanding the origin and the functional behavior of MEPs in normal and neoplastic mammary glands are of great importance due to the ongoing discussion regarding a tumor suppressor role of these cells, and the fact that partial MEP differentiation is often referred to as a basal cell phenotype, which is prominent in some aggressive triple negative breast cancers (TNBC) [4, 5]. Unfortunately, TNBC can be notoriously resistant to current therapies making it an urgent goal to identify novel therapeutic targets in this category of breast cancer.

The human breast gland is composed of branching epithelial ducts surrounded by a vascular-rich stroma [6, 7]. The epithelium is composed of two epithelial lineages, the inner layer of polarized LEPs and the outer layer of contractile MEPs [2, 8]. The human female breast gland is a unique organ, with its various developmental periods occurring first at puberty and later in adults. Through every estrous/menstruation cycle until the onset of menopause, continuous remodeling occurs [9]. In each cycle, there are ongoing phases of cell proliferation, differentiation, and apoptosis as the gland is preparing for pregnancy. If pregnancy occurs, there is a dramatic increase in cell proliferation to populate the vast number of lactating units needed for the offspring. During lactation, the gland reaches its terminal differentiation capacity. The proliferation of the MEPs needs to parallel that of the LEPs in the lobuli to secure efficient pumping of the milk through the duct system. During the lactating period, the suckling baby activates nerves in the nipple that results in the release of the contractile-inducing hormone oxytocin from the posterior pituitary. Oxytocin binds to the G-protein coupled oxytocin receptor expressed by MEPs leading to contraction of the cells through actin-myosin interactions and expulsion of the milk out of the duct system [10, 11]. After weaning, the glandular epithelium undergoes apoptosis, returning the gland to the similar pre-pregnancy state [12].

Although LEPs represent the functional (milk producing) cells in the mammary gland, they heavily rely on the MEPs for proliferation, differentiation, and luminal progenitor maturation [13•]. MEPs have been shown to directly regulate polarization and morphogenesis of the LEPs, and under normal conditions they can truly be called a guardian of epithelial integrity in the human breast gland [3, 14].

In recent years, researchers have recognized that the origin of breast cancer is linked to stem cells and different types of progenitor cells [15, 16] and many breast cancer subtypes have been shown to contain a partial myoepithelial expression pattern [4].

There are number of markers that discriminate between LEPs and MEPs. There is, however, to a certain degree, a blurred definition between basal cells and the myoepithelium. Often, basal cells and MEPs are used interchangeably but there is a justified need to identify markers that distinguish these two cell types as they have separate roles in breast biology.

In this review, we will discuss a number of topics centered on MEPs, including their cellular origin in the breast, their connection to basal cells and LEPs, and their heterotypic form and function during normal breast morphogenesis and neoplasia.

## Cellular Origin of Breast Myoepithelial Cells

Human breast development starts at 4 to 6 weeks of gestation [17], equivalent to embryonic days (E) 10.5 to E18.5 in mice [9]. Initially, a mammary ridge, or milk line, is formed in the ventral epidermis followed by invasion of the epidermal-derived epithelial cells into the underlying mesenchyme [12, 17]. The mesenchyme (hereafter referred to as the stroma) signals to the epithelium to invade and generate branching ducts. Interaction between the epithelium and stroma are crucial for maintenance and remodeling of the normal breast gland during adulthood, menstruation cycle, and pregnancy [6, 9].

Phenotypic characterization of the early invading epithelial buds in the developing human breast reveals two cell populations, centrally located epithelial cells and the peripherally located basal cells. The basal cells—that are considered precursors for myoepithelial cells—express CK17, p63, and vimentin but are negative for CK14 and alpha-smooth muscle actin (α-SMA), the ultimate differentiation marker for MEPs [18]. During fetal development, there are both spatial and temporal marker changes in basal cells, where cells acquire an increased myoepithelial differentiation pattern. It has been suggested that this plasticity within the basal cells relates to their ability to adapt to changes in the surrounding microenvironment [18, 19].

The most prominent markers for MEPs are basal cytokeratins such as CK14, CK5/6, and CK17. Other markers include P-cadherin, CD10, vimentin, and α-SMA, and although less frequently used, markers associated with fully differentiated myoepithelium including maspin (mammary serine protease inhibitor), calponin, and Wilms Tumor-1 (WT-1) [20, 21]. MEPs are the predominant cell type producing basement membrane matrix proteins such as laminins and collagen IV [22].

p63 is a member of the p53 family and a well-known stem cell and progenitor transcription factor for basal cells in stratified and pseudostratified epithelium such as the skin, prostate, esophagus, trachea, and bronchi [23–26]. Some of the p63-positive basally located cells in the breast gland (approx. 1 %) lack terminal myoepithelial differentiation, as determined by a lack of α-SMA expression [23]. Whether p63-positive, α-SMA-negative basal cells represent a precursor cell population is currently not known. Rios et al. demonstrated elegantly by in vivo labeling and cell tracing that bi-potential long-term cell populations could give rise to lineage-restricted LEPs and MEPs [27]. In this study, the Elf5 and CK5 were used as lineage markers for the LEPs and MEPs, respectively. Interestingly, the promoter for Elf5 is methylated in basal/myoepithelial cells [28], indicating the importance of epigenetic control in fate decision of breast epithelial cells.

Studies in rodents, particularly in mice, have paved the way for identification of potential stem cells in the human breast. Breast/mammary epithelial stem cells have been identified using cell labeling, cell sorting, in vitro assays, and transplantation into mammary cleared fat pads. Lineage tracing of cells in mice in vivo has been a successful method to identify cells with stem cell properties both spatially and temporally [9]. It is generally agreed upon that both LEPs and MEPs share a common stem or progenitor cell population, with a narrow marker profile including high expression of CD24, CD29, and CD49 [29, 30]. Most studies agree that stem or progenitor cells have a basal or suprabasal location. In line with this, Gudjonsson et al. described that suprabasal cells in the human breast that were MUC1-negative and EpCAM-positive contained progenitor or stem cell properties [31]. These cells were able to generate terminal duct lobular-like units (TDLUs) in 3D reconstituted basement membrane (3D-rBM) culture. Furthermore, an immortalized cell line, D492, derived from Muc1^neg^EpCAM^pos^ primary breast epithelial cells, is able to generate both LEPs and MEPs [31, 32•] (discussed below).

Prater et al. demonstrated recently that MEPs in mice are likely to represent the stem cell population in the mammary gland [33••]. They showed that distinct regions of myoepithelium expressing EpCAM were greatly enriched in mammary repopulating units (MRU). Interestingly, by inhibiting actin/myosin polymerization in MEPs, the MRU was greatly enhanced [33••], indicating that MEPs that lack actin and myosin contractility represent a cell population with increased stem cell activity or plasticity.

Two recent papers lend support to the importance of p63-positive basal cells as progenitors in tissue maintenance of the mammary gland. Chakrabarti et al. demonstrated that p63-positive basal cells retained stem cell properties through induction of Wnt signaling [34]. Moreover, Forster et al. showed that p63-positive basal cells provided instructive signals to LEPs during lactation. This was done via p63 transcriptional regulation of Nrg1, which in turn activated ErbB4/STAT5A in LEPs facilitating LEPs differentiation [13•].

## Double-Edged Role of Myoepithelial Cells in Normal and Neoplastic Breast Gland

Breast cancer rarely occurs in differentiated MEP [35]. In breast cancer, an unbroken layer of myoepithelium is considered a hallmark of cancer in situ [36]. Thus, for cancer cells to become invasive, they need to invade through the MEP layer, break down the basement membrane, and invade the surrounding stroma.

MEPs from normal breast and breast cancer differ in their ability to correctly form a basement membrane [3]. In this study, it was shown that MEPs in certain invasive breast cancers lack expression of alpha-1 chain of laminin-1, the major laminin isoform expressed in the breast. This lack of laminin-1 secretion is suggested to interfere with their ability to act as a tumor suppressor. It was also shown that MEPs isolated from normal breast could regulate polarization of LEPs when cultured in collagen gel. This was attributed to laminin-1 that was only expressed in MEPs from normal breast samples. Addition of pure laminin-1 could replace the MEPs in this assay and regulate polarization. Furthermore, it was shown that resident MEPs from invasive breast cancer failed to polarize LEPs in culture and that this was ascribed to the absence of laminin-1. Indeed, laminin-1 is one of the major components of Matrigel (a reconstitutive basement membrane matrix) that is widely used for culture of breast epithelium due to its power to induce differentiation, including polarization of LEPs.

Thus, it is important when discussing the tumor suppressor function of MEPs, that we realize that full acquisition of myoepithelial differentiation is most likely required to see the full tumor suppressor properties of these cells. Gene expression analysis and phenotypic characterization of breast cancers has identified many subclasses that can be related to various progenitor cells during the stepwise lineage development of both luminal and myoepithelial cells [37]. This supports the hypothesis that the cell-of-origin for each category can be traced to different progenitor cell populations during breast cancer development. Interestingly, many tumors bearing worse prognoses and showing EMT-like phenotypes co-express several markers common to normal MEPs. P-cadherin is upregulated in triple negative breast cancer (TNBC) including basal-like and metaplastic cancers [38]. This cadherin is expressed in MEPs, and also in stem and progenitor cells of the breast [38].

Epithelial to mesenchymal transition (EMT) is a developmental event important for branching morphogenesis in the mammary gland but is also important for cancer cell invasion and progression [39, 40]. Petersen et al. previously showed that cancer cells generate their own non-malignant stroma through EMT [41]. These cancer cells were derived from a metaplastic breast cancer of the TNBC subtype that exhibited mixed expression of luminal epithelial, myoepithelial, and mesenchymal markers [42]. Although non-tumorigenic, these fibroblastic-like cells facilitated cancer growth both in in vitro assays and in mice. This demonstrates that cancer-derived stroma, although non-tumorigenic, can be a facilitator of tumor progression [41].

## In Vitro Models of Human Breast Morphogenesis

In vivo models have contributed tremendously to our understanding of mammary gland morphogenesis and neoplasia. There is, however, a considerable difference between animal models and humans in terms of breast mammary gland histology and cell-cell interactions. Therefore, cell culture models that reproducibly capture essential features of human breast morphogenesis and cancer progression are desirable, and those pre-existing models have proven their worth (reviewed by [43, 44].

Three-dimensional (3D) cell cultures in either reconstituted basement membrane (rBM) or collagen have been useful in capturing phenotypic traits of in vivo breast morphogenesis [45]. Culturing breast-derived cells in 3D-rBM has been useful to discriminate between normal cells and cancer cells [46]. In 3D-rBM culture, LEPs are able to form correctly polarized acini. In contrast, cancer-derived epithelial cells fail to do so under similar conditions [46]. This is just one example where the culture conditions allow for the distinction between normal and cancerous epithelium, with maintenance of the respective histoarchitecture. In the same assay, MEPs form compact non-polarized spheres, making them easily identifiable in culture. In 3D collagen assays, LEPs form acinus-like structures with inversed polarity, indicating that the necessary microenvironment present in rBM is lacking in the collagen gel. This polarity can be rescued by addition of MEPs. In co-culture with LEPs, MEPs secrete the basement membrane product laminin-1, which induces correct polarity in LEPs [3].

The 3D-rBM assay has been useful to study branching morphogenesis and EMT. D492 is a breast epithelial cell line with stem cell properties that contains phenotypic traits for both LEPs and MEPs when cultured in monolayer, and in 3D-rBM, they form branching structures reminiscent of TDLUs [31]. We have recently generated a heterotypic co-culture model where endothelial cells have been shown to stimulate growth and morphogenesis of breast epithelial cells [47]. In another study, we demonstrated that endothelial cells stimulate both growth and branching morphogenesis of D492 cells. Surprisingly, we observed that a subset of D492 cells underwent EMT in co-culture with endothelial cells to form mesenchymal structures, which we later isolated and established a cell subline referred to as D492M [32•]. D492M cells exhibit marker expression of mesenchymal cells, namely an E- to N-cadherin switch, downregulation of keratins, and upregulation of EMT transcription factors. MicroRNA (miR) analysis of D492 and D492M showed that a number of miRs are differentially regulated. One of the most profound changes was the downregulation of the miR-200 family. More recently, we have shown that ectopic expression of miR-200c-141 in D492M induced a mesenchymal-to-epithelial transition (MET) with luminal epithelial cell differentiation. These cells lacked, however, the potential to form TDLUs in 3D-rBM. When we overexpressed p63 in the mesenchymal D492M cells, the cells showed signs of myoepithelial differentiation, with increased expression of P-cadherin and basal cytokeratins such as CK5/6 and 17 [48]. Similar to the D492^miR-200-141^ overexpressing D492M cells, the p63 overexpressing D492M cells failed to form TDLU-like structures. When both miR-200c-141 and p63 are co-expressed in D492M cells, the cells gain both luminal and myoepithelial phenotypes as measured by their marker expression. Moreover, in 3D culture, the cells recapitulate the TDLU-like branching structures seen in the parental D492 cells [48]. Thus, in a histoarchitectural context, both the luminal and MEP are dependent on each other to build the correct histological structures necessary to maintain a structural and functional gland.

Recently, Linnemann et al. established a powerful 3D collagen assay to study branching morphogenesis of primary breast epithelial cells [49•]. This assay is based on culturing primary breast epithelial cells in collagen, which is then detached from the tissue culture plate resulting in a floating cellular gel in the given culture medium. Linnemann and coworkers demonstrated that basal cells expressing the CD10 antigen were the cells that could repeatedly generate TDLU-like structures with an inner layer of luminal-like cells and an outer layer of contractile MEP. This and previous studies demonstrate that cells with partial myoepithelial differentiation are able to capture the bi-layered branching morphogenesis of breast epithelial ducts in culture.

In a recent paper by Hines et al., it was shown that in primary culture, basal/myoepithelial cells but not luminal epithelial cells were the preferred target cells for lentiviral-based immortalization [50]. Furthermore, they revealed that the mucin barrier of the luminal cells protects them against bacterial and viral infections; hence, making them more resistant to transgenic lentiviral vector transduction. Pre-treating LEPs with neuraminidase reduced the mucin barrier and increased the effectiveness of luminal epithelial transduction [50]. This could explain the difficulty experienced in establishing cell lines with a pure luminal differentiation profile. Indeed, most cell lines, although originally believed to be of luminal origin, have a basal phenotype.

## Myoepithelial Pathology and its Link to Invasion and EMT

Recent findings showing the myoepithelium as an active participator in cancer progression have shifted pathologists’ attention towards the differentiation status of the myoepithelium. The WHO classification of breast tumors has thus put more focus on the involvement of MEPs in breast cancer [51]. Neoplasia composed of MEPs is referred to as malignant myoepithelioma. These tumors are categorized as metaplastic tumors with EMT phenotype, as assessed by spindle-like cells with positive expression of α-SMA, similar to MEPs [51]. Also, these tumors often contain a mixture of LEPs and MEPs. Mixed population of cells with partial LEPs and MEPs differentiation is found in many tumors of the TNBC lineages such as the aforementioned metaplastic cancer, and basal-like cancer [52].

In the literature, there is a vague separation between basal cells and MEPs. The terms are often used interchangeably and sometimes basal cells are referred to as stem cells, or as a cell type within certain breast cancer subtypes, such as basal-like breast cancer. It will be important in the near future to define clearly the biological differences between basal cells and MEPs to aid clarification of the various breast cancer subtypes.

## Discussion

**Fig. 1:**
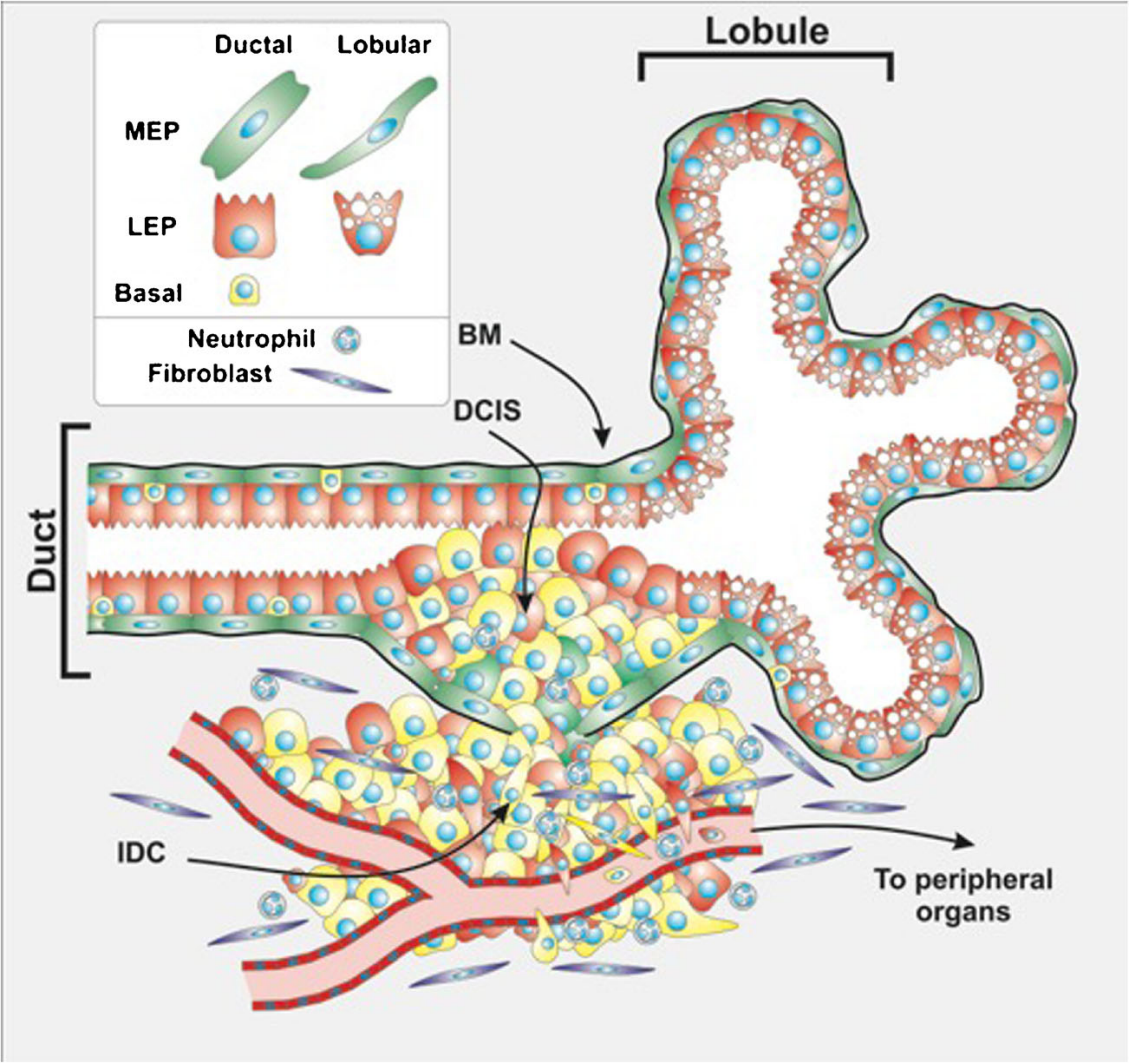
Schematic figure showing TDLU and cancer progression in the breast. The mammary gland is composed of a double epithelial cell layer of myoepithelial cells (*green cells*) and luminal epithelial cells (*red cells*). In ducts, MEPs fully enclose the LEPs, isolating them from the surrounding stroma. In lobuli, MEPs are thinner and form a more discontinuous basket-like structure, meaning that some LEPs come in contact with the basement membrane (*BM*, *black line*). Within the epithelial layer, basal progenitors can be found (*yellow cells*). These cells share characteristics of both luminal and myoepithelial cells. During breast cancer progression, lesions develop in situ. These lesions are surrounded by a layer of MEPs and basement membrane. When lesions progress into invasive cancer, the myoepithelial cells are lost, and the basement membrane is broken. Basal cancer consists of a heterogeneous cell population, often poorly differentiated; these tumors rarely have differentiated myoepithelial cells, but often they have acquired mesenchymal characteristics, having undergone EMT (*elongated red/yellow cells*). EMT cells can enter capillaries and lymphatic vessels, leading to distant metastases. Stromal fibroblasts (*blue*) and neutrophils (*gray*) often infiltrate the tumor and the surrounding stroma

In this review, we have discussed the symbiotic interaction between LEPs and MEPs within the human breast gland, with particular focus on the MEPs. MEPs play an important role as keepers of luminal epithelial integrity in the breast, both by providing signals for luminal epithelial differentiation and by producing and maintaining the basement membrane matrix separating the epithelium from the surrounding stroma. In generic terms, cancer is thought to initiate via proliferation of epithelial cells that later acquire properties enabling them to invade through the myoepithelial barrier, break down the basement membrane matrix, and migrate into the vascular-rich stroma before entering the blood or lymphatic vessels and disseminating to distant organs. The phenotypic switch from epithelial to mesenchymal is one of the weapons cancer cells use to invade and migrate through the matrix. Previously, MEPs were largely neglected in the discussion of breast cancer formation and progression but this has been rapidly changing in recent years. MEPs express many phenotypic traits of mesenchymal cells and partial myoepithelial differentiation is a prominent profile in many TNBCs. It is important to discriminate between partially differentiated and fully differentiated myoepithelium. The latter can be defined as terminally differentiated and non-proliferative cells that play an important guarding role in the normal breast. The partially differentiated myoepithelial cells, on the other hand, are likely to represent the different stages of progenitor cells arrested in a partially differentiated state (Fig. 1).

Myoepithelial cells are, as we have discussed in this review, key players in normal differentiation and maintenance of the human breast gland. It is, however, becoming increasingly clear that cells with partial myoepithelial differentiation can shift roles to be facilitators of cancer progression, either through EMT or as a non-malignant part of the tumor stromal environment that fuels the cancer cells with growth factors and extracellular matrix facilitating their growth.
